# Life-threatening Development of Cardiac Tamponade in the Span of 24 Hours

**DOI:** 10.5811/cpcem.2019.4.42741

**Published:** 2019-07-01

**Authors:** Patrick Kishi, Thaer Ahmad, Kenneth W. Dodd

**Affiliations:** Advocate Christ Medical Center, Department of Emergency Medicine, Oak Lawn, Illinois

## Abstract

Cardiac tamponade is a medical emergency that requires immediate treatment. Caused by the development of fluid in the pericardial space, it can result in a severe decrease in cardiac output. When encountering patients with severe hypotension and tachycardia, emergency physicians must always consider the diagnosis of tamponade to facilitate prompt and effective treatment and stabilization. We report our experience with a patient who developed life-threatening cardiac tamponade within a span of less than 24 hours.

## INTRODUCTION

Cardiac tamponade is a medical emergency that is caused by compression of the cardiac chambers due to an accumulation of fluid in the pericardial space.^1^,^2^ Approximately 15–30 milliliters (mL) of fluid lies between the parietal pericardium and visceral pericardium in normal physiologic states.^3^,^4^ If fluid accumulation surpasses a critical threshold, cardiac output and blood pressure drop precipitously.^4^,^5^ However, due to the adaptive and elastic properties of the pericardium, the amount of pericardial fluid that can be tolerated is related to the rate of the accumulation of fluid.^1^ Chronic accumulation of fluid gives the pericardium time to gradually increase its compliance; thus, tamponade may not be seen until a large amount of fluid has developed in the pericardial sac. In acute settings, the pericardium does not have time to increase its compliance, leading to a rapid decline in cardiac output.

Several causes of cardiac tamponade have been identified, including idiopathic, infectious, autoimmune, malignancy, trauma, and metabolic etiologies.^6^–^8^ One study identified the most common diagnoses of patients with pericardial effusions as follows: acute idiopathic pericarditis (20%), iatrogenic cause (16%), malignancy (13%), chronic idiopathic effusion (9%), acute myocardial infarction (8%), end stage renal disease (6%), and tuberculosis or bacterial infection (4%).^4^,^9^

The clinical presentation of cardiac tamponade can vary widely. Acute presentations of tamponade can occur within minutes or hours, as is typically seen in trauma, aortic rupture, or iatrogenic result from an invasive procedure. Acute presentations frequently result in life-threatening hypotension necessitating immediate interventions to decrease the pericardial pressure.^10^ In contrast, the subacute presentations can occur over days to weeks and hypotension is relatively uncommon.^4^,^11^ One study demonstrated that 27–43% of patients presenting with subacute cardiac tamponade are actually hypertensive, with systolic blood pressures ranging from 127 millimeter of mercury (mmHg) to 144 mmHg, likely secondary to increased activity of the sympathetic nervous system.^11^,^12^ Clinical findings of tamponade can vary, and no sensitive or specific signs have been identified.^13^ Findings may include tachycardia, jugular venous distension, pulsus paradoxus, tachypnea, hypotension, hypertension, and pericardial rub. The classic presentation of Beck’s triad, characterized by hypotension, increased jugular venous pressure, and muffled heart sounds, is seen only in a small number of acute cardiac tamponade cases.^14^

When considering cardiac tamponade in patients, the diagnostic workup should include an electrocardiogram, chest radiograph, and echocardiogram. While tamponade is mostly a clinical diagnosis, echocardiography is highly recommended by multiple task forces and societies.^13^,^15^ If available in the emergency department (ED), point-of-care ultrasound should be used by emergency physicians to help identify and possibly treat acute cardiac tamponade.

Our report describes a life-threatening presentation of cardiac tamponade that developed over the span of one day in a young, female patient.

## CASE REPORT

A 23-year-old African-American female presented to our ED with one day of sharp, midline chest pain that radiated to her jaw and left arm. She reported mild associated shortness of breath but no exertional symptoms. The review of systems was otherwise unremarkable. Her past medical history was significant for adrenal insufficiency on daily hydrocortisone, hypothyroidism, and two prior episodes of pericardial effusion with previous drainage procedures. Vital signs included a blood pressure of 129/96 mmHg, heart rate 99 beats per minute, and oxygen saturation 100% on room air. The patient was afebrile. Initial laboratory workup was unremarkable with a negative troponin. Due to the patient’s history of pericardial effusions, a formal transthoracic echocardiogram was performed, which demonstrated normal systolic function with an ejection fraction of 50–55% and no evidence of a pericardial effusion (Video 1). The patient was then discharged home.

The following day, the patient presented to our ED with similar symptoms, including chest pain and shortness of breath. Upon evaluation, her vital signs demonstrated severe hypotension with a blood pressure of 71/52 mmHg, heart rate 121 beats per minute, respiratory rate 16 breaths per minute, oxygen saturation 100% on room air, and temperature 36.8°C. Her exam was now significant for depressed mental status and lethargy. A point-of-care cardiac ultrasound was performed, which demonstrated a large amount of pericardial fluid and right ventricular collapse. Immediate transthoracic echocardiogram confirmed these findings (Videos 2 and 3).

Interventional cardiology and cardiovascular surgery were consulted, and a decision was made to take the patient to the operating room emergent pericardiectomy. In the operating room, approximately 300 mL of turbid, yellow fluid was evacuated from the underlying pericardium. A 24 French Blake drain was placed in the pericardium and the patient was admitted to the intensive care unit.

With the patient’s previous pericardial effusion, past laboratory workup demonstrated high titers for coxsackie B virus. Upon this admission, a viral panel was negative, including hepatitis B panel, influenza, respiratory syncytial virus, corona virus, metapneumovirus, parainfluenza virus types 1,3, and 4, Epstein-Barr virus, cytomegalovirus, human immunodeficiency virus, coxsackie, influenza A, myocoplasma pneumonia, *Chlamydia pneumoniae*, buccal virus, and rhinovirus. Rheumatologic workup was also negative, including antinuclear antibody, anti-double stranded deoxyribonucleic acid, antimicrosomal antibody, anticentromere antibody, Sjogren’s syndrome A and B, Smith antibody, celiac screen, anti-Jo 1 antibody, and anti-SCL–70 antibody (anti-topoisomerase I). Further testing for anti-21 alpha hydroxylase and anti-TPO antibodies were also negative.

**CPC-EM Capsule**

What do we already know about this clinical entity?

Cardiac tamponade is a life-threatening illness that requires emergent recognition and treatment.

What makes this presentation of disease reportable?

We present a unique case of a patient who developed severe, life-threatening cardiac tamponade in under 24 hours that necessitated immediate intervention.

What is the major learning point?

Cardiac tamponade can develop rapidly, and recent negative imaging does not rule it out. Point-of-care ultrasound can help rapidly identify this disease process.

How might this improve emergency medicine practice?

Emergency physicians must always consider cardiac tamponade in hypotensive, tachycardic patients.

Pericardial fluid analysis demonstrated predominantly acute inflammatory cells without any evidence of malignancy. Pericardial biopsy results were unrevealing. Repeat echocardiogram demonstrated normal left ventricular ejection fraction of 50–55% and no significant pericardial effusion. The patient was discharged 10 days following initial presentation and has not had any subsequent episodes of cardiac tamponade or pericardial effusions diagnosed at our institution.

## DISCUSSION

Our report highlights a unique presentation of life-threatening, atraumatic cardiac tamponade that developed over the span of 24 hours in a young female with a history of pericardial effusion.

Echocardiography has a class I recommendation to evaluate most pericardial diseases as it can identify anatomic, physiologic, and hemodynamic abnormalities of the heart.^15^ When evaluating for pericardial effusions, this imaging modality can estimate the size and location of the effusion. Effusions can be classified based on quantitative measurements: small <10 mm, moderate 10–20 mm, and large >20 mm in size.^13^ Echocardiographic signs of tamponade include collapsing cardiac chambers, changes in chamber volumes and flows with respiration, and inferior venous cava dilation.^13^ While any heart chambers may collapse in tamponade, the most common findings are right-sided atrial and ventricular collapse, and studies have demonstrated that right atrial collapse is highly sensitive and specific for tamponade if it persists for longer than one-third of the cardiac cycle.^15^ Collapse of the right ventricle is less sensitive than right atrial collapse, but very specific for tamponade.^16^

This patient was treated by a cardiothoracic surgeon who performed a pericardial window to remove the pericardial fluid. Most patients with cardiac tamponade require drainage of the effusion to improve symptoms. The two methods of removal include percutaneous drainage by a pericardiocentesis and surgical drainage by creating a pericardial window (pericardiectomy). While each method has its advantages, our patient underwent surgical pericardiectomy. This procedure allows the clinician to obtain pericardial biopsies for further evaluation, which was an important consideration in this patient given her history of recurrent effusions. For our patient, analysis of the fluid and subsequent rheumatologic and malignant workups were negative. Interestingly, the patient tested negative for the most common etiologies of tamponade. Upon discharge, a repeat echocardiogram demonstrated no evidence of re-accumulation of a pericardial effusion.

## CONCLUSION

Despite the acute, life-threatening presentation of cardiac tamponade, this patient was successfully treated by surgical pericardiectomy to remove pericardial fluid. While there are many known causes of cardiac tamponade, our patient had a completely negative workup. This case illustrates the importance for emergency physicians to always consider the diagnosis of cardiac tamponade when encountering patients with severe hypotension and tachycardia. Without prompt and proper identification of this diagnosis, patients have a high likelihood of experiencing severe morbidity and possibly mortality. Immediate point-of-care cardiac ultrasound and transthoracic echocardiography can aid in the diagnosis. In conclusion, we report a unique presentation of severe cardiac tamponade that developed within a 24-hour interval period and was successfully treated with a pericardial window.

## Supplementary Information

Video 1. A formal parasternal long-axis view of the heart in the patient when she initially presented to the emergency department. No significant pericardial effusion is seen.

Video 2. A formal parasternal long-axis view of the heart of the patient when she re-presented the following day. A large pericardial effusion can be seen. (Arrows indicate surrounding fluid).

Video 3. A formal apical four-chamber view of the heart demonstrating a large pericardial effusion (Top arrow demonstrates fluid, bottom arrow demonstrates right atrial collapse).
